# Difluorinated Cyclohexanes: Energetics and Intra‐ and Intermolecular Interactions

**DOI:** 10.1002/cphc.202500648

**Published:** 2025-10-10

**Authors:** Matheus P. Freitas

**Affiliations:** ^1^ Department of Chemistry, Institute of Natural Sciences Federal University of Lavras Lavras 37200‐900 MG Brazil

**Keywords:** density functional theory calculations, difluorocyclohexanes, electrostatics, hyperconjugation, steric effects

## Abstract

Fluorinated cyclohexanes are essential building blocks for high‐performance molecules, including ion carriers and liquid crystals. This quantum‐chemical study evaluates the relative energies of all difluorinated cyclohexane isomers, highlighting key intramolecular interactions as thermodynamic modulators. The stability of 1,1‐difluorocyclohexane arises from an anomeric‐like *n*_F_ → σ*_CF_ interaction, despite F—C—F repulsion. Dipolar repulsion between C—F bonds affects isomers like diequatorial *trans*‐1,2 and diaxial *cis*‐1,3, while axial C—H···C—F electrostatic attraction stabilizes *trans*‐1,3 and diaxial *trans*‐1,4 isomers. Hyperconjugation involving antiperiplanar C—H and C—F bonds also contributes. Notably, diaxial C—F bonds facilitate self‐assembly via interactions between negatively charged fluorines and positively charged hydrogens. This study advances understanding of fluorinated cyclohexanes’ thermodynamic behavior, providing a framework for designing tailored fluorinated compounds for advanced applications.

## Graphical abstract

This quantum‐chemical study examines all difluorocyclohexane isomers, revealing how intramolecular interactions govern stability. Key factors include n_F_ → σ*_CF_ hyperconjugation in 1,1‐isomers, C—F dipolar repulsion, C—H···C—F electrostatic attraction, and antiperiplanar hyperconjugation. Diaxial C—F bonds promote self‐assembly, guiding the design of advanced fluorinated compounds.

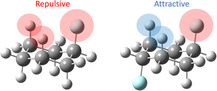

## 1 Introduction

Organofluorines play a pivotal role in chemistry, with applications ranging from fragrance compounds and liquid crystals to pharmaceuticals, agrochemicals, and high‐performance materials such as Teflon.^[^ ^1^, ^2^, ^3^, ^4^, ^5^, ^6^, ^7^, ^8^ ^]^ The carbon–fluorine (C—F) bond profoundly influences the physicochemical properties of these compounds due to its high polarity, exceptional bond strength, and the relatively small size of the fluorine atom.^[^ ^9^, ^10^ ^]^ In particular, fluorine substitution in organic molecules significantly affects conformational equilibria, often altering molecular behavior compared to the corresponding nonfluorinated alkanes.^[^ ^11^ ^]^

One of the most emblematic examples of fluorine's conformational influence is the *gauche effect* observed in vicinal difluorinated compounds.^[^ ^12^ ^]^ This phenomenon is primarily attributed to a subtle stereoelectronic interaction between antiperiplanar σ_C__–__H_ and σ*_C__–__F_ orbitals in ethane‐like fragments, favoring the *gauche* conformation over the *anti* one.^[^ ^13^, ^14^, ^15^, ^16^ ^]^ Although 1,2‐difluoroethane exhibits a clear preference for the *gauche* conformer, this trend does not universally apply. For instance, *trans*‐1,2‐difluorocyclohexane adopts a diaxial conformation that is more stable than its diequatorial counterpart, despite the latter placing the fluorine atoms in a *gauche*‐like arrangement.^[^ ^17^ ^]^

Freitas and coworkers^[^ ^18^ ^]^ investigated the conformational preferences of various difluorinated cyclohexanes—including 1,2‐, 1,3‐, and 1,4‐substituted systems—and found that their stability is governed by distinct electrostatic and steric factors: dipolar repulsion in the diequatorial conformer of *trans*‐1,2‐difluorocyclohexane; *syn*‐1,3‐diaxial repulsion in the diaxial conformer of *cis*‐1,3‐difluorocyclohexane; and attractive electrostatic interactions between axial hydrogens and fluorines in the diaxial conformer of *trans*‐1,4‐difluorocyclohexane. These findings were benchmarked against the conformational behavior of fluorocyclohexane. Additionally, Luo and coworkers^[^ ^19^ ^]^ determined the relative energies of fluorinated cyclohexanes and developed a predictive model for the energies of difluorocyclohexanes. However, their study did not interpret the relative stabilities in terms of specific intramolecular interactions.

**Figure 1:**
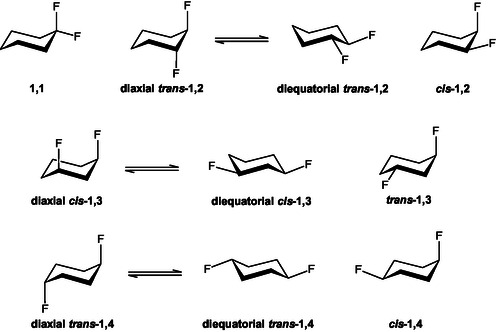
Difluorinated cyclohexanes studied herein through quantum‐chemical calculations.

In the present quantum‐chemical study, this analysis is extended by exploring both the conformational and constitutional stabilities of all possible isomers of difluorocyclohexane: 1,1‐, *cis*‐1,2‐, *trans*‐1,2‐, *cis*‐1,3‐, *trans*‐1,3‐, *cis*‐1,4‐, and *trans*‐1,4‐difluoro derivatives (**Figure** **1**). An isodesmic comparison was employed to evaluate their relative energetics. Furthermore, to gain deeper insight into the nature of the interactions governing their stability, a natural bond orbital (NBO) analysis was conducted. This included an assessment of both Lewis‐type (classical bonding) and non‐Lewis‐type (electron delocalization) contributions to the total electronic energy of each system. The findings offer a detailed understanding of how the position and spatial orientation of fluorine atoms shape the conformational preferences and regiochemistry of a prototypical cycloalkane‐like cyclohexane. These insights may prove valuable in the rational design of molecules where fluorine positioning and conformation play critical roles in defining their properties.

## 2 Computational Methods

The difluorocyclohexanes in Figure 1 were fully optimized using density functional theory (DFT) at the B3LYP‐GD3BJ/6‐311++G(d,p) level,^[^ ^20^, ^21^, ^22^ ^]^ which includes dispersion corrections for improved accuracy (though their effect was minimal; see Supporting Information).^[^ ^23^ ^]^ Calculations were performed for both the gas phase and implicit DMSO using the Solvation Model Density (SMD).^[^ ^24^ ^]^ The results were consistent with those from a benchmark study, as the observed trends in relative energies were in agreement across the levels tested (Supporting Information), confirming the reliability of this approach. The dimers of diaxial conformations were computed at the same level of theory, including counterpoise correction, starting from structures in which the fluorines were oriented toward the axial hydrogens. This computational approach has demonstrated reliability in predicting the energies and properties of organofluorine compounds.^[^ ^25^ ^]^ Frequency calculations were performed to confirm the absence of imaginary frequencies and to derive standard Gibbs free energies and enthalpies. Natural bond orbital (NBO) analysis was conducted using the NBO 7.0 program^[^ ^26^ ^]^ to identify electron donor‐acceptor interactions contributing to electron delocalization within the systems. The NOSTAR/NBODEL keyword was employed to calculate deletion energies by removing unoccupied (antibonding and Rydberg) orbitals, offering insights into the non‐Lewis contributions (electron delocalization) and their role alongside the Lewis‐type (classical bonding) component in the total electronic energy. All calculations were carried out using the Gaussian 16 software suite.^[^ ^27^ ^]^ The atoms‐in‐molecules (AIM) analyses were performed using the AIMAll program.^[^ ^28^ ^]^

## 3 Results and Discussion

**Figure 2:**
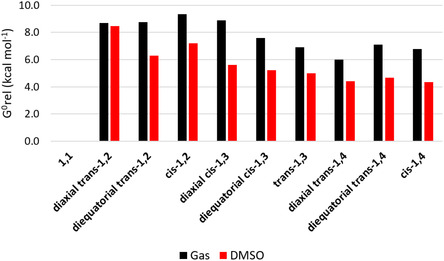
Relative Gibbs free energies (kcal mol^−1^) of difluorocyclohexanes.

The 10 structures depicted in Figure 1 were investigated using quantum chemical methods to determine their relative energies and to understand their stabilities (**Figure** **2**). The energy trends observed in the gas phase remain largely consistent in implicit DMSO, with a few exceptions. Notably, highly polar structures are more affected by solvation. For example, the most polar compound, **diaxial** ***cis*** **‐1,3**, is significantly stabilized in the polar medium. While energetics plays a crucial role in defining the conformational equilibrium of compounds such as *trans*‐1,2‐, *cis*‐1,3‐, and *trans*‐1,4‐difluorocyclohexane, it is less directly relevant for evaluating the relative stability of regioisomers and configurational stereoisomers. For example, all‐*cis*‐1,2,3,4,5,6‐hexafluorocyclohexane, although the least stable isomer among 1,2,3,4,5,6‐hexafluorocyclohexanes,^[^ ^29^ ^]^ can still be synthesized readily via the catalytic hydrogenation of hexafluorobenzene.^[^ ^30^ ^]^ However, landscaping the overall energetic patterns of difluorinated cyclohexanes provides valuable insights into the favorable positions and orientations for fluorination along the hydrocarbon skeleton. Additionally, it sheds light on the intramolecular interactions unique to each system. To this end, the energetics of stereoisomers and constitutional isomers will be analyzed separately and interpreted in terms of intramolecular interactions, supported by natural bond orbital analyses and the quantum theory of atom‐in‐molecules.However, prior to the NBO analyses, the following isodesmic reaction can be useful for assessing the effect—whether stabilizing or destabilizing—of incorporating an additional fluorine atom into fluorocyclohexane. This provides insight into possible intramolecular interactions between the fluorine atoms or other interactions involving them.

(1) 2 Fluorocyclohexane → Cyclohexane + Difluorocyclohexane

**Table** **1** shows that only two processes are exothermic: the formation of the 1,1‐isomer (by 6.4 kcal mol^−^^1^) and the diaxial *trans*‐1,4‐isomer (by 0.3 kcal mol^−^^1^). The least favorable reaction is the formation of ***cis*** **‐1,2**, followed by **diequatorial** ***trans*** **‐1,2**, **diaxial** ***cis*** **‐1,3**, and **diaxial** ***trans*** **‐1,2**. This trend clearly indicates that proximity between C—F bonds reduces stability, either through direct steric contact or weaker, remote electronic interactions. For example: a) **diequatorial** ***trans*** **‐1,2** suffers from *gauche* repulsion between polar C—F bonds; b) **diaxial** ***cis*** **‐1,3** is destabilized by *syn*‐1,3‐diaxial interactions between the C—F bonds; c) **diaxial** ***trans*** **‐1,2** exhibits antiperiplanar σ_CF_ → σ*_CF_ interactions, in contrast to the more stabilizing σ_CH_ → σ*_CF_ interactions found in fluorocyclohexane.

**Table 1:** Enthalpy changes (kcal mol^−1^) for the isodesmic conversion of two fluorocyclohexane molecules (either axial or equatorial, depending on the resulting difluorinated product) into difluorocyclohexane and cyclohexane.

Product of the isodesmic reaction	Δ*H* ^0^
**1,1**	−6.39
**diaxial *trans*‐1,2**	2.45
**diequatorial *trans*‐1,2**	2.88
***cis*‐1,2**	3.18
**diaxial *cis*‐1,3**	2.69
**diequatorial *cis*‐1,3**	1.69
***trans*‐1,3**	0.85
**diaxial *trans*‐1,4**	−0.27
**diequatorial *trans*‐1,4**	1.20
***cis*‐1,4**	0.71

The ***cis*** **‐1,2** isomer features one axial and one equatorial C—F bond in a *gauche* arrangement. Its formation is less favorable than that of **diequatorial** ***trans*** **‐1,2**, likely due to the presence of an axial C—F bond, and also less favorable than **diaxial** ***trans*** **‐1,2**, as the *gauche* orientation of the C—F bonds appears to be more destabilizing than the presence of two axial C—F bonds.

Interestingly, the formation of ***trans*** **‐1,3** is less disfavored than that of **diequatorial** ***cis*** **‐1,3**, and the formation of ***cis*** **‐1,4** is less disfavored than that of **diequatorial** ***trans*** **‐1,4**, despite both ***trans*** **‐1,3** and ***cis*** **‐1,4** having an axial C—F bond. This suggests that not all axial C—F bonds are equally destabilizing. While in fluorocyclohexane, *syn*‐1,3‐diaxial interactions between C—H and C—F bonds destabilize the axial conformer, in difluorinated cyclohexanes, this interaction may be less pronounced. The increased acidity of axial C—H bonds may give rise to attractive C—F···H—C contributions, mitigating the destabilization. To better understand these effects, a natural bond orbital (NBO) analysis is presented.

### 3.1 Stereoisomers

*Trans*‐1,2‐, *cis*‐1,3‐, and *trans*‐1,4‐difluorocyclohexanes undergo chair interconversion, generating diaxial and diequatorial conformers. In the *trans* isomers, the diaxial conformer is favored, while the diequatorial conformer is more stable in *cis*‐1,3‐difluorocyclohexane. This preference suggests that fluorine atoms repel each other when positioned on the same side, consistent with steric and dipolar considerations. However, despite experiencing *syn*‐1,3‐diaxial interactions with hydrogens—commonly repulsive, as observed in methylcyclohexane and fluorocyclohexane (which favor equatorial conformations)—the *trans* isomers prefer the diaxial conformation.

Interestingly, *trans*‐1,2‐difluorocyclohexane does not exhibit the *gauche* effect seen in 1,2‐difluoroethane, even with the *gauche* arrangement of C—F bonds. To elucidate these behaviors, a natural bond orbital (NBO) analysis was performed, decomposing the total electronic energy (E) into Lewis‐type (E_L_) and non‐Lewis‐type (E_NL_) terms, as defined in Equation (2). The E_L_ term reflects classical interactions, including Pauli repulsion, while the E_NL_ term accounts for stabilizing electron delocalization between donor and acceptor orbitals. In the context of NBO theory, a Lewis structure is constructed under the assumption of perfectly localized electrons—bonding and non‐bonding orbitals are fully occupied, while antibonding orbitals have zero occupancy. As a result, Lewis‐type interactions occur exclusively between fully occupied orbitals and are, therefore, inherently repulsive.

(2) $$E=E_{L}+E_{NL}$$

**Figure 3:**
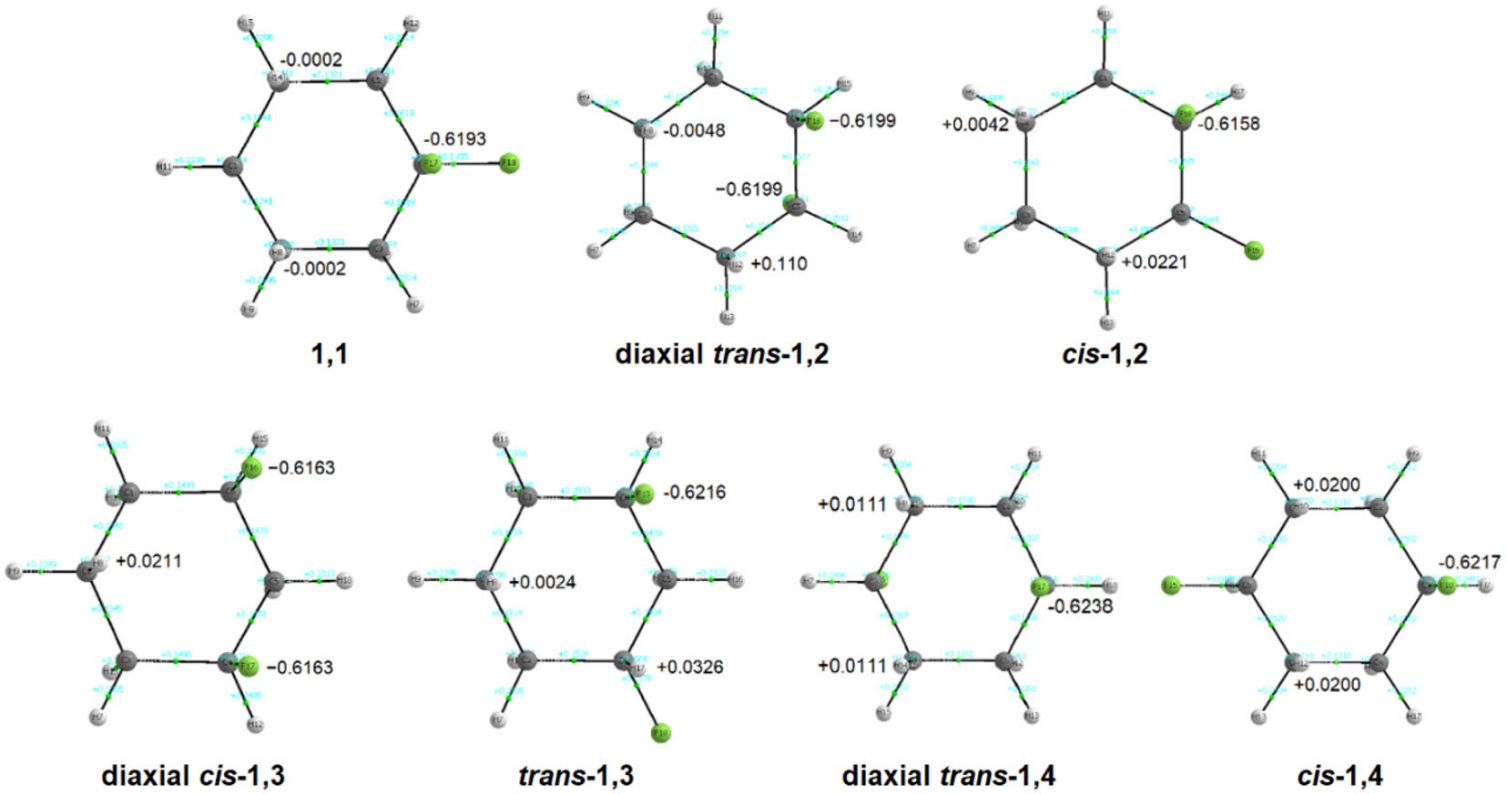
QTAIM plots showing atomic charges for axial fluorines and interacting hydrogens.

**Table** **2** indicates that the diaxial conformer of *trans*‐1,2‐difluorocyclohexane experiences weaker steric repulsion compared to the diequatorial conformer but benefits less from stabilizing electron delocalization effects. Since the E_L_ term predominates, the diaxial conformer is slightly favored. Unlike 1,2‐difluoroethane, where stabilizing hyperconjugation offsets dipolar *gauche* repulsion, the diequatorial conformer of *trans*‐1,2‐difluorocyclohexane lacks antiperiplanar C—H bonds necessary for this effect. Instead, the less effective electron donor C–C bond occupies this position, further diminishing stabilization. Compared to the ***cis*** **‐1,2** stereoisomer, both *trans*‐1,2 conformers have lower overall energies, despite the greater electron delocalization stabilization observed in the *cis* isomer. This is primarily due to the *gauche* arrangement of fluorines in *cis*‐1,2‐difluorocyclohexane, which induces significant dipolar repulsion. Moreover, the axial C—F bond in the *cis* isomer experiences 1,3‐diaxial interactions with hydrogens, further increasing the E_L_ term and destabilizing the structure. Although these 1,3‐diaxial interactions may include an attractive electrostatic component—arising from the negative fluorine and positively charged axial hydrogens on the same side (as shown in the QTAIM plots of **Figure** **3**)—the repulsive contribution ultimately predominates. This is because, in ***cis*** **‐1,2**, only one axial C—H bond exhibits a significantly positive hydrogen capable of contributing to such attraction.

**Table 2:** Relative Gibbs free energies (gas/implicit DMSO), electronic conformational energies, Lewis and non‐Lewis contributions to the electronic energies of difluorocyclohexanes (in kcal mol^−1^), and molecular dipole moments (in D).

Compound	G^0^_rel_	E_rel_	E_L_	E_NL_	μ
**1,1**	0.00/0.00	0.00	322.8	−322.8	2.91
**diaxial *trans*‐1,2**	8.70/8.47	8.62	293.4	−284.8	0.98
**diequatorial *trans*‐1,2**	8.77/6.30	8.80	299.4	−290.6	3.88
***cis*‐1,2**	9.35/7.20	9.21	299.8	−290.6	3.46
**diaxial *cis*‐1,3**	8.90/5.62	8.95	300.0	−291.1	3.87
**diequatorial *cis*‐1,3**	7.58/5.21	7.54	297.0	−289.5	2.62
***trans*‐1,3**	6.92/4.99	6.86	295.3	−288.4	2.51
**diaxial *trans*‐1,4**	5.99/4.41	5.88	294.9	−289.0	0.00
**diequatorial *trans*‐1,4**	7.09/4.67	7.04	296.5	−289.5	0.00
***cis*‐1,4**	6.79/4.36	6.72	297.0	−290.3	3.06

In the conformational equilibrium of *cis*‐1,3‐difluorocyclohexane, the methylene group separating the C—F bonds increases the energy gap in favor of the diequatorial conformer, compared to *trans*‐1,2‐difluorocyclohexane. This occurs because, in the transition from *trans*‐1,2 to *cis*‐1,3, the diequatorial fluorines move farther apart and cease to interact significantly, whereas the axial fluorines adopt a *syn*‐1,3‐diaxial orientation, leading to stronger unfavorable interactions. Hyperconjugation (E_NL_) favors both the diequatorial *trans*‐1,2 (by 5.8 kcal mol^−1^) and the diaxial *cis*‐1,3 (by 1.6 kcal mol^−1^) conformers relative to their alternatives. Therefore, the enhanced energy difference between the diaxial and diequatorial conformers in *cis*‐1,3‐difluorocyclohexane is primarily attributed to the E_L_ term (by 3.0 kcal mol^−1^). However, the diaxial conformer is partially stabilized by about 1.5 kcal mol^−^^1^ due to electron delocalization effects. Interestingly, the *trans*‐1,3 stereoisomer is more stable than either conformer of *cis*‐1,3‐difluorocyclohexane, despite having an axial C—F bond (!), due to its lower overall Lewis‐type energy.

Intriguingly, the diaxial conformer of *trans*‐1,4‐difluorocyclohexane is ≈1 kcal mol^−^^1^ more stable than the diequatorial conformer, primarily due to reduced E_L_ destabilization. This raises the question: Why would diametrically opposed C—F bonds in a cyclohexane ring favor the axial orientation, given that fluorocyclohexane predominantly adopts an equatorial conformation? Despite QTAIM calculations showing no bond path between axial fluorines and 1,3‐positioned hydrogens, each negatively charged fluorine (−0.6217) can interact with two highly positive hydrogens (+0.0200). These interactions form electrostatic C—H···F—C hydrogen bonds, totaling four interactions in the *trans*‐1,4 diaxial conformer, supporting an earlier explanation of Wiberg et al.^[^ ^31^ ^]^ The higher positive charge on these hydrogens results from their proximity to the C—F bonds, which enhances their acidity. In contrast, the *cis*‐1,4 stereoisomer allows only two such interactions, resulting in intermediate stability. Although the diaxial *trans*‐1,4 isomer is not the most favored by E_NL_, it experiences a double hyperconjugation, where the interaction is extended to involve not just one σ‐bond, but two σ‐bonds on adjacent carbons.^[^ ^32^ ^]^

### 3.2 Positional Isomers

In general, the stability of positional isomers increases as the spacing between C—F bonds grows, except for 1,1‐difluorocyclohexane, which is significantly stabilized by electron delocalization. A detailed analysis of donor‐acceptor interactions reveals that 1,1‐difluorocyclohexane gains substantial stabilization from two anomeric‐like interactions, specifically *n*_F_ → σ*_CF_, contributing 14.5–15.0 kcal mol^−1^ each.

Notably, despite the exception, the most stable structure features two axial C—F bonds (*trans*‐1,4‐difluorocyclohexane), contrary to expectations based on systems like dimethylcyclohexane. While fluorine is smaller than a methyl group but larger than hydrogen, repulsive *syn*‐1,3‐diaxial interactions might be anticipated. Moreover, the highly polar nature of the C—F bond suggests potential dipolar repulsion with another C—F bond in *gauche* or *syn*‐1,3 arrangements. Conversely, interactions with oppositely polarized bonds can be attractive. As a result, not all structures with at least one axial C—F bond are unfavorable, as demonstrated by the diaxial *trans*‐1,2‐difluorocyclohexane, *trans*‐1,3‐difluorocyclohexane, and diaxial *trans*‐1,4‐difluorocyclohexane. In these cases, high stability arises from reduced Lewis‐type interactions, which reflect a combination of minimized Pauli repulsion and favorable electrostatic effects. In contrast, fluorocyclohexane exhibits a markedly different behavior, preferring the equatorial C—F orientation. A plausible explanation for the equatorial preference in fluorocyclohexane is that its axial hydrogens are less acidic than those in the difluorinated derivatives, making *syn*‐1,3‐diaxial interactions predominantly repulsive and thus disfavoring the axial orientation of the C—F bond.

A methylene spacer increases the distance between polar C—F bonds, contributing to stabilization. For instance, the most stable stereoisomer of 1,2‐difluorocyclohexane is less stable than that of 1,3‐difluorocyclohexane, which, in turn, is less stable than the diaxial *trans*‐1,4‐difluorocyclohexane. However, this stabilization is not solely due to the reduced interactions between polar C—F bonds as their separation increases. While increasing the bond distance can attenuate dipolar repulsion—such as in the comparison between the diequatorial conformers of *trans*‐1,2‐ and *trans*‐1,4‐difluorocyclohexanes—it can also enhance such interactions, as observed when comparing the diaxial conformers of *trans*‐1,2‐ and *cis*‐1,3‐difluorocyclohexanes. It is important to note that in these systems, particularly those with axial C—F bonds, attractive and repulsive electrostatic interactions coexist with σ_CH_ → σ*_CF_ hyperconjugation effects.

As a general observation, interacting C—F bonds exhibit strong repulsion, surpassing the *syn*‐1,3‐diaxial interactions typically observed with hydrogens. The latter interactions can, in fact, be attractive, as demonstrated by notable examples. For instance, *trans*‐1,3‐difluorocyclohexane is lower in energy than its diequatorial *cis*‐1,3‐difluorocyclohexane counterpart, and the diaxial *trans*‐1,4‐difluorocyclohexane is more stable than its diequatorial conformer.

### 3.3 Intermolecular Interactions

**Figure 4:**
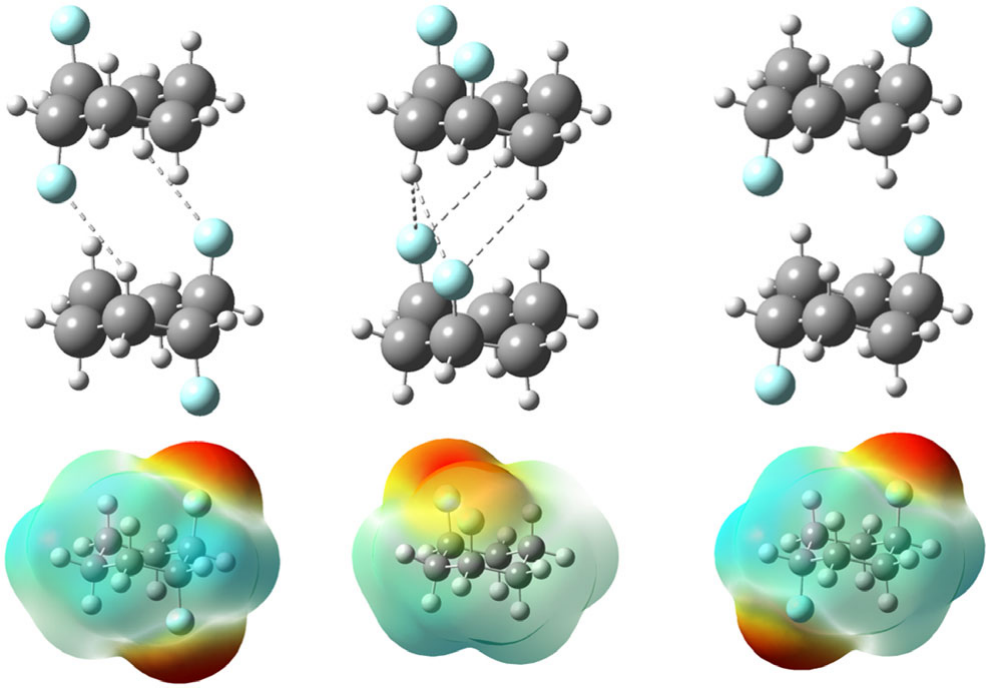
Self‐assembly of difluorocyclohexanes with both C—F bonds in axial orientation, along with the electrostatic potential surface.

The most polar and least stable isomer of 1,2,3,4,5,6‐hexafluorocyclohexane, the all‐*cis* form, exhibits a Janus‐like polarized face. This configuration allows interaction with ions and self‐assembly through the alignment of axial fluorines (negative face) with axial hydrogens (positive face).^[^ ^33^ ^]^ Similarly, the highly polar **diaxial** ***cis*** **‐1,3** isomer (3.87 D) can electrostatically associate with another analogous molecule, offering insights into its likely supramolecular structure in the condensed phase (**Figure** **4**). This association is thermodynamically (Δ*H* ^0^) favorable by 4.0 kcal mol^−^^1^, as demonstrated by comparing the enthalpy of the dimer to the sum of the enthalpies of the two isolated species.

**Figure 5:**
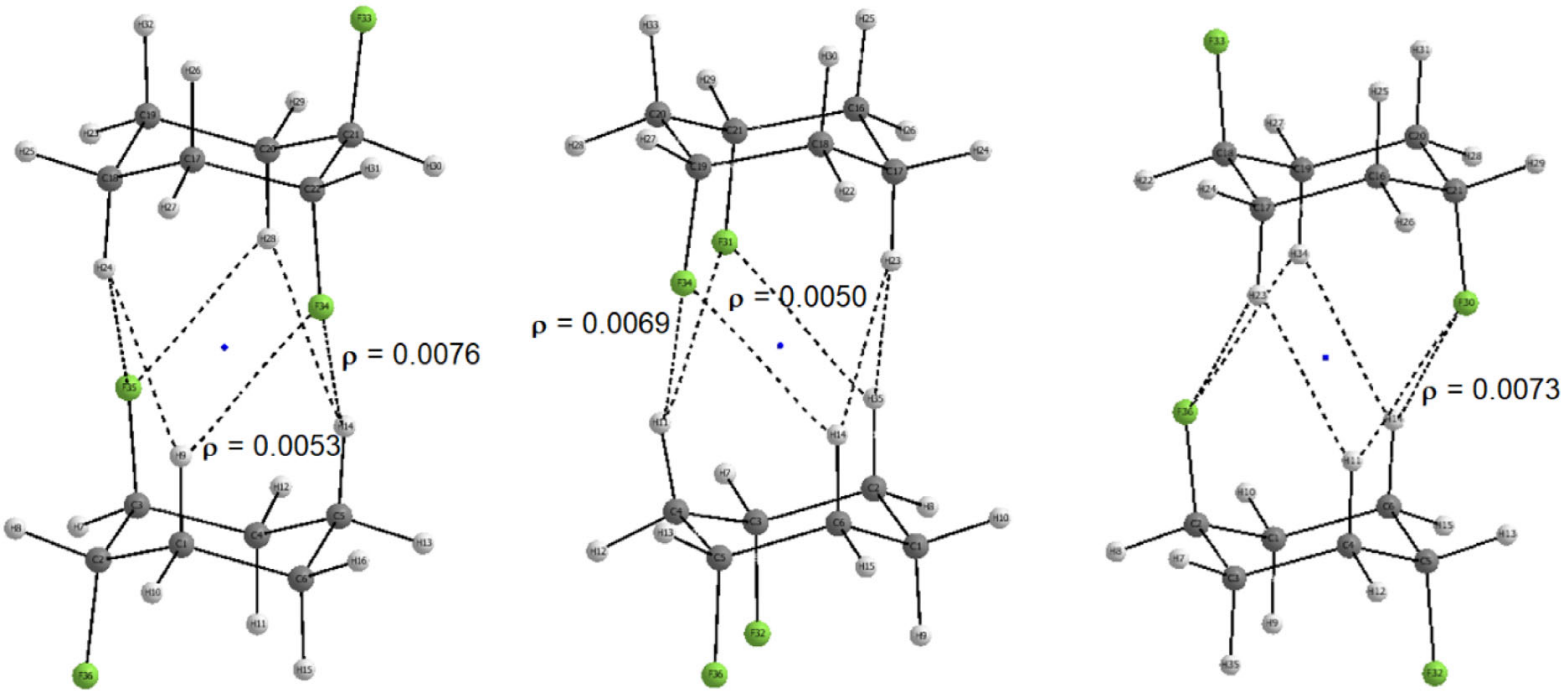
QTAIM plots showing bond paths and electron densities (ρ) at the bond critical points for the dimers of **diaxial** ***trans*** **‐1,2**, **diaxial** ***cis*** **‐1,3**, and **diaxial** ***trans*** **‐1,4** structures.

Interestingly, the other two diaxial isomers, ***trans*** **‐1,2** and ***trans*** **‐1,4**, while nearly nonpolar, also form stable dimers with intermolecular interaction energies of −2.5 and −3.6 kcal mol^−^^1^, respectively. Among these structures, **diaxial** ***cis*** **‐1,3** exhibits the highest potential for self‐assembly, as the axial H‐2 is more acidic due to its proximity to both C—F bonds, enhancing its interaction with the fluorines of another molecule. QTAIM analysis (**Figure** **5**) reveals that these interactions, characterized by bond paths and electron densities at bond critical points, can be described as electrostatic hydrogen bonds.

## 4 Conclusion

This study provides a comprehensive analysis of the conformational preferences and energetics of mono‐ and difluorinated cyclohexanes, offering insights into how fluorine substitution influences molecular behavior. In general, fluorocyclohexane favors the equatorial conformation of the C—F bond over the axial one. However, introducing a second fluorine atom significantly alters the system by increasing the acidity of neighboring hydrogens and promoting interactions between polar C—F bonds. These subtle but important effects play a crucial role in shaping the stability of different conformers and can ultimately influence the self‐assembly of fluorinated structures.

The conformational trends of difluorocyclohexanes reveal distinct interaction patterns depending on the substitution pattern. In 1,3‐ and 1,4‐difluorocyclohexanes, axial C—F bonds engage in favorable electrostatic interactions with axial C—H bonds, partially offsetting the syn‐1,3‐diaxial repulsion that usually dominates the equilibrium of fluorocyclohexane. In contrast, *trans*‐1,2‐difluorocyclohexane exhibits strong repulsion between diequatorial C—F bonds. Notably, the gauche effect is absent in this system due to the lack of antiperiplanar C—H and C—F bonds, whose stabilizing interactions are more favorable than those involving antiperiplanar C–C and C—F bonds.

The influence of distance between fluorine atoms was also observed. While increasing the number of intervening methylene groups generally reduces repulsion between C—F bonds, certain configurations—such as the diaxial *cis*‐1,3‐difluorocyclohexane—bring the fluorine atoms closer together, enhancing dipolar repulsion compared to structures like the diaxial *trans*‐1,2‐difluorocyclohexane.

Overall, these findings establish clear structure–interaction relationships for fluorinated cyclohexanes. By elucidating how the position and orientation of C—F bonds dictate conformational stability, this work provides a fundamental framework for the rational design of organofluorine compounds with targeted properties, including materials such as liquid crystals.

## Supporting Information

Benchmark results, standard coordinates and absolute energies of the optimized geometries.

## Conflict of Interest

The author declares no conflict of interest.

## Author Contributions

**Matheus P. Freitas**: conceptualization (lead); formal analysis (lead); funding acquisition (lead); investigation (lead); methodology (lead); project administration (lead); writing—original draft (lead); writing—review and editing (lead).

## Supporting information

Supplementary Material

## Data Availability

The data that support the findings of this study are available in the supplementary material of this article.

## References

[1] R. Callejo , M. J. Corr , M. Yang , M. Wang , D. B. Cordes , A. M. Z. Slawin , D. O’Hagan , Chem. – Eur. J. 2016, 22, 8137. 27149882 10.1002/chem.201600519

[2] M. J. Corr , R. A. Cormanich , C. N. von Hahmann , M. Bühl , D. B. Cordes , A. M. Z. Slawin , D. O’Hagan , Org. Biomol. Chem. 2016, 14, 211. 26584449 10.1039/c5ob02023a

[3] P. T. Lowe , D. O’Hagan , J. Fluorine Chem. 2020, 230, 109420.

[4] D. O’Hagan , Chem. – Eur. J. 2020, 26, 7981.32083392

[5] D. O’Hagan , R. J. Young , Med. Chem. Res. 2023, 32, 1231.

[6] H.‐J. Böhm , D. Banner , S. Bendels , M. Kansy , B. Kuhn , K. Müller , U. Obst‐Sander , M. Stahl , ChemBioChem 2004, 5, 637. 15122635 10.1002/cbic.200301023

[7] Y. Ogawa , E. Tokunaga , O. Kobayashi , K. Hirai , N. Shibata , iScience 2020, 23, 101467. 32891056 10.1016/j.isci.2020.101467 PMC7479632

[8] T. Fujiwara , D. O’Hagan , J. Fluorine Chem. 2014, 167, 16.

[9] D. O’Hagan , Chem. Soc. Rev. 2008, 37, 308.18197347

[10] L. Hunter , Beilstein J. Org. Chem. 2010, 6, 38. 20502650 10.3762/bjoc.6.38 PMC2874311

[11] P. Ryan , R. Iftikhar , L. Hunter , Beilstein J. Org. Chem. 2025, 21, 680. 40196389 10.3762/bjoc.21.54 PMC11973591

[12] S. Wolfe , Acc. Chem. Res. 1972, 5, 102.

[13] L. Goodman , H. Gu , V. Pophristic , J. Phys. Chem. A 2005, 109, 1223. 16833433 10.1021/jp046290d

[14] D. Y. Buissonneaud , T. van Mourik , D. O’Hagan , Tetrahedron 2010, 66, 2196.

[15] F. A. Martins , M. P. Freitas , Eur. J. Org. Chem. 2019, 2019, 6401.

[16] D. R. Silva , L. A. Santos , T. A. Hamlin , C. F. Guerra , M. P. Freitas , F. M. Bickelhaupt , ChemPhysChem 2021, 22, 641. 33555663 10.1002/cphc.202100090 PMC8048458

[17] K. B. Wiberg , W. Hinz , R. M. Jarret , R. K. B. Aubrecht , J. Org. Chem. 2005, 70, 8381. 16209581 10.1021/jo051049w

[18] M. P. Freitas , C. F. Tormena , P. R. Oliveira , R. Rittner , J. Mol. Struct.: Theochem 2002, 589–590, 147.

[19] Q. Luo , K. R. Randall , H. F. Schaefer , RSC Adv. 2013, 3, 6572.

[20] A. D. Becke , Phys. Rev. A 1988, 38, 3098. 10.1103/physreva.38.3098 9900728

[21] C. Lee , W. Yang , R. G. Parr , Phys. Rev. B 1988, 37, 785. 10.1103/physrevb.37.785 9944570

[22] R. Ditchfield , W. J. Hehre , J. A. Pople , J. Chem. Phys. 1971, 54, 724.

[23] S. Grimme , S. Ehrlich , L. Goerigk , J. Comput. Chem. 2011, 32, 1456. 21370243 10.1002/jcc.21759

[24] A. V. Marenich , C. J. Cramer , D. G. Truhlar , J. Phys. Chem. B 2009, 113, 6378. 19366259 10.1021/jp810292n

[25] M. P. Freitas , J. Fluorine Chem. 2025, 282, 110397.

[26] E. D. Glendening , J. K. Badenhoop , A. E. Reed , J. E. Carpenter , J. A. Bohmann , C. M. Morales , P. Karafiloglou , C. R. Landis , F. Weinhold , F. NBO 7.0, Theoretical Chemistry Institute, University of Wisconsin, Madison 2018.

[27] M. J. Frisch , G. W. Trucks , H. B. Schlegel , G. E. Scuseria , M. A. Robb , J. R. Cheeseman , G. Scalmani , V. Barone , G. A. Petersson , H. Nakatsuji , X. Li , M. Caricato , A. V. Marenich , J. Bloino , B. G. Janesko , R. Gomperts , B. Mennucci , H. P. Hratchian , J. V. Ortiz , A. F. Izmaylov , J. L. Sonnenberg , D. Williams‐Young , F. Ding , F. Lipparini , F. Egidi , J. Goings , B. Peng , A. Petrone , T. Henderson , D. Ranasinghe , et al., Gaussian 16, Revision C.01, Gaussian, Inc., Wallingford CT 2016.

[28] T. A. Keith , AIMAll (Version 19.10.12), TK Gristmill Software, Overland Park KS, USA 2019.

[29] N. S. Keddie , A. M. Z. Slawin , T. Lebl , D. Philp , D. O’Hagan , Nat. Chem. 2015, 7, 483. 25991526 10.1038/nchem.2232

[30] M. P. Wiesenfeldt , Z. Nairoukh , W. Li , F. Glorious , Science 2017, 357, 908. 28798044 10.1126/science.aao0270

[31] K. B. Wiberg , W. Hinz , R. M. Jarret , K. B. Aubrecht , J. Org. Chem. 2005, 70, 8381. 16209581 10.1021/jo051049w

[32] I. V. Alabugin , M. Manoharan , J. Org. Chem. 2004, 69, 9011. 15609933 10.1021/jo048287w

[33] D. O´Hagan , Chem. Rec. 2023, 23, e202300027. 37016509 10.1002/tcr.202300027

