# Supplementary material for: Difluorinated Cyclohexanes: Energetics and Intra‐ and Intermolecular Interactions
Source: Chemphyschem. 2025 Oct 10;26(23):e202500648. doi: 10.1002/cphc.202500648 (PMC12677719; doi:10.1002/cphc.202500648)
Supplement: Supplementary file 1 — Supplementary Material [file CPHC-26-e202500648-s001.pdf]

SUPPORTING INFORMATION

Difluorinated Cyclohexanes: Energetics, Intra- and Intermolecular Interactions

Matheus P. Freitas[a]*

[a] Department of Chemistry, Institute of Natural Sciences, Federal University of Lavras, 37200-900, Lavras, MG, Brazil

* E-mail: matheus@ufla.br

Page S2: Figure S1. Benchmark study comparing different levels of calculations.

Pages S3-S14: Optimized geometries of difluorinated cyclohexanes with their corresponding Gibbs free energies (in hartrees), calculated at the B3LYP-GD3BJ/6-311++G(d,p) level of theory.

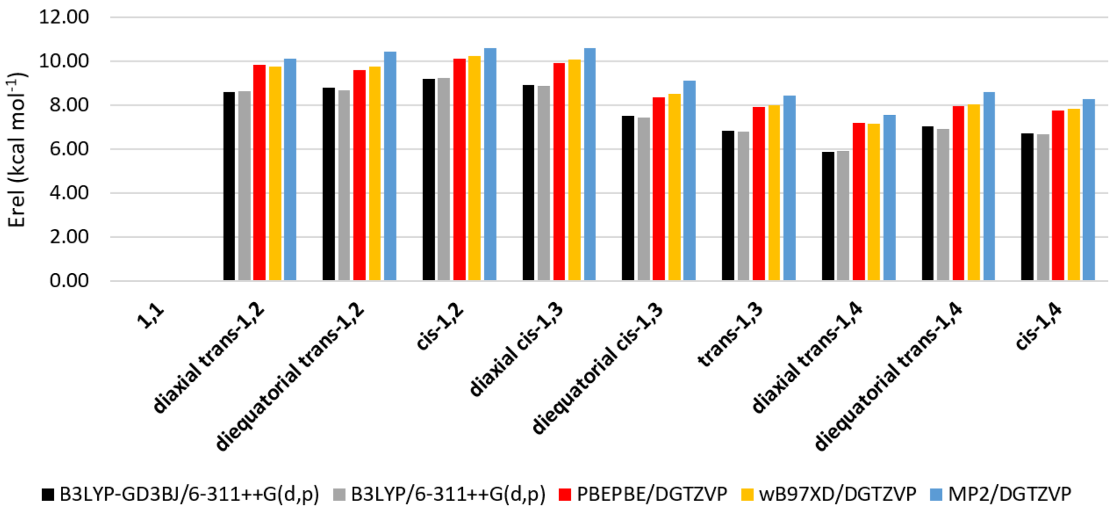

Figure S1. Benchmark study comparing different levels of calculations.

1,1-Difluorocyclohexane (gas phase)

0 1

C 2.02796100 0.00000400 -0.23713300

C 1.26700200 1.26613800 0.17675200

C -0.15708500 1.27387600 -0.39674900

C -0.89450400 -0.00000400 -0.03664000

C -0.15708400 -1.27388900 -0.39675600

C 1.26700200 -1.26613200 0.17675000

H -0.73798200 2.12317800 -0.03016000

H 1.21408500 1.31961800 1.26888700

H 1.79802300 2.16068700 -0.15904800

H 2.16945600 0.00000500 -1.32539800

H 3.02614900 -0.00000500 0.20970000

H -0.73797100 -2.12318700 -0.03014300

H -0.12671400 -1.33618600 -1.48893100

H 1.21407300 -1.31960100 1.26888400

H 1.79804100 -2.16067700 -0.15903300

H -0.12670900 1.33616200 -1.48892700

F -1.14908900 -0.00000400 1.33008900

F -2.14093500 0.00000900 -0.63933000

G^0 = -434.405315 hartrees

Diaxial *trans*-1,2-difluorocyclohexane (gas phase)

0 1

C -0.35481600 1.10852900 -0.97885600

C -1.61763700 0.73940200 -0.18993200

C -1.61443700 -0.74631400 0.18994200

C -0.35004400 -1.11002600 0.97885100

C 0.91754500 -0.72510700 0.23675400

C 0.91441900 0.72903600 -0.23676300

H -2.49964400 -0.98968300 0.78376000

H -1.66858200 1.35052900 0.71726700

H -2.50389000 0.97894700 -0.78374200

H -0.34293800 0.57543200 -1.93582900

H -0.32367100 2.17786100 -1.20413900

H -0.34043000 -0.57689500 1.93583000

H -0.31431500 -2.17922000 1.20411800

H 1.80878100 -0.91456800 0.83901700

H 1.80482700 0.92234100 -0.83903200

H -1.66275900 -1.35764300 -0.71726400

F 1.04062600 -1.52014000 -0.92720200

F 1.03409100 1.52455900 0.92720700

G^0 = -434.391447 hartrees

H^0 = -434.351783 hartrees

Diequatorial *trans*-1,2-difluorocyclohexane (gas phase)

0 1

C 0.60296300 -1.46817800 -0.10024000

C 1.87155800 -0.70482700 0.30101400

C 1.87155100 0.70483300 -0.30101000

C 0.60295500 1.46818100 0.10023800

C -0.65352900 0.70025700 -0.29000000

C -0.65352100 -0.70025500 0.28999500

H 2.75429800 1.26194200 0.02332800

H 1.92928900 -0.63364200 1.39378200

H 2.75430600 -1.26192600 -0.02333700

H 0.58717000 -1.62023500 -1.18528300

H 0.56915800 -2.45724700 0.36249600

H 0.58715300 1.62024200 1.18528100

H 0.56916100 2.45725100 -0.36249800

H 1.92928100 0.63364000 -1.39377800

F -1.77799500 -1.39814900 -0.18376000

F -1.77800500 1.39813700 0.18376300

H -0.76384700 0.63876700 -1.37752600

H -0.76383700 -0.63875400 1.37752100

G^0 = -434.391337 hartrees

Cis-1,2-difluorocyclohexane (gas phase)

0 1

C 1.07950100 -1.08802800 -0.67326600

C 1.90271500 -0.10888700 0.17570100

C 1.36969800 1.32440800 0.05353700

C -0.12788900 1.39791300 0.39412200

C -0.90359400 0.42812100 -0.48020900

C -0.40525600 -1.00042000 -0.33860900

H 1.92910200 1.99453900 0.71131700

H 1.86117000 -0.42464500 1.22241900

H 2.95222200 -0.15132300 -0.12798900

H 1.20448700 -0.86271300 -1.73913700

H 1.41383200 -2.11742900 -0.52306900

H -0.29318800 1.12987600 1.44115800

H -0.51685000 2.40774500 0.24381400

H 1.52870800 1.68718500 -0.96995600

F -2.27148000 0.48082200 -0.16876200

H -0.81727600 0.70966400 -1.53626900

H -1.00810900 -1.67402300 -0.95231300

F -0.58909200 -1.41610200 0.99569200

G^0 = -434.390416 hartrees

Diaxial *cis*-1,3-difluorocyclohexane (gas phase)

0 1

C -1.26132000 1.11437800 0.21059300

C -0.00004800 1.56936400 -0.53087700

C 1.26123100 1.11445500 0.21059300

C 1.28470700 -0.39446400 0.41295200

C 0.00000500 -0.93783900 1.03091000

C -1.28470000 -0.39454100 0.41293300

H 2.16467100 1.41067000 -0.32825800

H -0.00003100 1.14734800 -1.53901100

H -0.00007200 2.65718200 -0.63767400

H -1.30641000 1.58824200 1.19935300

H -2.16476300 1.41052000 -0.32828200

H 2.14165000 -0.68855000 1.02575100

H 1.30628000 1.58829800 1.19936400

H 0.00000800 -0.66516200 2.09301000

H -2.14166800 -0.68871000 1.02571800

F -1.48899700 -1.00886300 -0.84285200

F 1.48911400 -1.00880000 -0.84281300

H 0.00003400 -2.02899100 0.97838700

G^0 = -434.391130 hartrees

H^0 = -434.351413 hartrees

Diequatorial *cis*-1,3-difluorocyclohexane (gas phase)

0 1

C 1.26966000 1.06600500 -0.23279700

C 0.00000000 1.82237900 0.18585900

C -1.26967200 1.06600500 -0.23276600

C -1.24915300 -0.34624300 0.32803700

C 0.00000400 -1.11112200 -0.09026400

C 1.24915200 -0.34623200 0.32803400

H -2.16632600 1.58632900 0.11258700

H 0.00001400 1.96549000 1.27330500

H -0.00000900 2.82134300 -0.25559200

H 1.32587200 1.00278500 -1.32489200

H 2.16632700 1.58633400 0.11251400

H -1.32592600 1.00280700 -1.32486000

H 0.00000400 -1.22267400 -1.17907300

H 0.00000600 -2.10960300 0.35197200

H -1.33509500 -0.32121900 1.42015700

F -2.38210500 -1.04973400 -0.12905900

F 2.38211500 -1.04972800 -0.12904100

H 1.33508900 -0.32118500 1.42015300

G^0 = -434.393237 hartrees

***Trans*-1,3-difluorocyclohexane (gas phase)**

0 1

C -1.48906300 0.74915900 0.58605200

C -0.57871300 1.61435100 -0.29402600

C 0.90223400 1.28358100 -0.05641700

C 1.15364000 -0.19528500 -0.29542400

C 0.27440400 -1.07734400 0.57966500

C -1.20112300 -0.73525400 0.41287100

H 1.54538400 1.87529400 -0.71243900

H -0.82943700 1.44525400 -1.34585900

H -0.76152700 2.67282000 -0.09332000

H -1.33455000 0.99772800 1.64280500

H -2.54247700 0.93564600 0.36234200

H 1.18109700 1.52099100 0.97687500

H 0.55197400 -0.91908600 1.62750500

H -1.81334900 -1.33617700 1.08948600

F -1.59506400 -1.11385500 -0.89807800

H 0.44073600 -2.13135600 0.34736600

H 1.00938300 -0.44245500 -1.35038000

F 2.50445200 -0.49213400 -0.00644500

G^0 = -434.394282 hartrees

Diaxial *trans*-1,4-difluorocyclohexane (gas phase)

0 1

C 0.52061300 1.26619900 -0.56337900

C -0.52059200 1.26623100 0.56331600

C -1.36384000 -0.00008700 0.55686900

C -0.52062300 -1.26624200 0.56306200

C 0.52070400 -1.26622700 -0.56313400

C 1.36378600 -0.00006400 -0.55678400

H -2.07538500 -0.00019200 1.38623600

H -0.01648500 1.32839400 1.53265400

H -1.17411800 2.13791200 0.48006600

H 0.01640800 1.32822600 -1.53268400

H 1.17406000 2.13794800 -0.48025800

H 0.01684200 -1.32860400 -1.53290800

H 1.17412200 -2.13821900 -0.48020500

H -0.01679600 -1.32865400 1.53285300

H -1.17408200 -2.13823000 0.48014100

F -2.15634800 0.00013600 -0.62504400

F 2.15630000 0.00016100 0.62511700

H 2.07557600 -0.00011300 -1.38625800

G^0 = -434.395775 hartrees

H^0 = -434.356124 hartrees

Diequatorial *trans*-1,4-difluorocyclohexane (gas phase)

0 1

C 0.74667600 1.26153900 -0.18020500

C -0.74667300 1.26155000 0.18017900

C -1.41995500 -0.00000500 -0.33861800

C -0.74663700 -1.26151100 0.18015000

C 0.74664000 -1.26151300 -0.18017900

C 1.41994200 -0.00001000 0.33861700

H -0.87516300 1.29677200 1.26778300

H -1.24110400 2.14376000 -0.23232800

H 0.87516200 1.29673600 -1.26781100

H 1.24112000 2.14375400 0.23227800

H 0.87519700 -1.29675300 -1.26774100

H 1.24109600 -2.14372400 0.23218500

H -0.87519000 -1.29676900 1.26771200

H -1.24110200 -2.14371500 -0.23222200

H 1.43493500 0.00002000 1.43414400

H -1.43499200 0.00003000 -1.43414500

F -2.77098600 -0.00002500 0.06763000

F 2.77099400 -0.00002100 -0.06757700

G^0 = -434.394017 hartrees

***Cis*-1,4-difluorocyclohexane (gas phase)**

0 1

C -0.57952100 1.25930700 0.08975700

C 0.80231400 1.26731900 -0.58078000

C 1.58985100 -0.00001400 -0.27812900

C 0.80229400 -1.26733700 -0.58076800

C -0.57953400 -1.25930500 0.08976100

C -1.34327300 0.00000700 -0.28489000

H 2.54559900 -0.00002700 -0.80866100

H 0.69440400 1.34162000 -1.66954900

H 1.37872200 2.13744600 -0.25835800

H -0.46418600 1.28519000 1.17687100

H -1.15188400 2.14271400 -0.20301500

H -0.46422400 -1.28517000 1.17684800

H -1.15189800 -2.14267300 -0.20301100

H 0.69440000 -1.34162500 -1.66950900

F -2.58789900 0.00000400 0.38073300

H -1.57842300 0.00002000 -1.35604400

H 1.37868000 -2.13744200 -0.25835200

F 1.91745700 0.00000600 1.10183100

G^0 = -434.394500 hartrees

1,1-difluorocyclohexane (DMSO – SMD)

0 1

C -2.03136300 0.00000000 -0.23554200

C -1.27248000 -1.26512800 0.18046400

C 0.15257100 -1.27455800 -0.39116800

C 0.88351000 0.00000000 -0.04268700

C 0.15257100 1.27455800 -0.39116700

C -1.27248000 1.26512800 0.18046400

H 0.73227800 -2.12471000 -0.02292400

H -1.22705400 -1.32220100 1.27335100

H -1.79774700 -2.16009300 -0.16263300

H -2.16701100 0.00000000 -1.32406600

H -3.02966100 -0.00000100 0.21144600

H 0.73227800 2.12471000 -0.02292400

H 0.11697500 1.33551300 -1.48284200

H -1.22705500 1.32220200 1.27335100

H -1.79774700 2.16009300 -0.16263300

H 0.11697500 -1.33551300 -1.48284200

F 1.17673400 0.00000000 1.32889700

F 2.14257700 0.00000100 -0.65106100

G^0 = -434.41502 hartrees

Diaxial *trans*-1,2-difluorocyclohexane (DMSO – SMD)

0 1

C 0.36212100 1.11706400 0.96977600

C 1.62436700 0.74302100 0.18393900

C 1.62361400 -0.74467100 -0.18392800

C 0.36099000 -1.11743500 -0.96976800

C -0.91145300 -0.72239000 -0.24784400

C -0.91071600 0.72331800 0.24783800

H 2.50701000 -0.99044400 -0.77950400

H 1.68070900 1.34517900 -0.72967900

H 2.50801000 0.98790000 0.77951800

H 0.35707200 0.59619800 1.93387100

H 0.32535100 2.18790300 1.18816400

H 0.35648000 -0.59656800 -1.93386600

H 0.32313300 -2.18823600 -1.18815200

H -1.79847000 -0.90980000 -0.85426500

H -1.79755000 0.91163700 0.85424500

H 1.67934400 -1.34688600 0.72968900

F -1.05827500 -1.53157200 0.92222800

F -1.05668300 1.53264600 -0.92224000

G^0 = -434.401533 hartrees

***Cis*-1,2-difluorocyclohexane (DMSO – SMD)**

0 1

C 1.06977100 -1.10862400 -0.65535300

C 1.90320700 -0.14085900 0.19492700

C 1.40179400 1.30063900 0.05437200

C -0.09552400 1.40797900 0.38653200

C -0.87843600 0.44934800 -0.48803700

C -0.41627200 -0.98741000 -0.36122700

H 1.96733800 1.96676200 0.71100400

H 1.85414100 -0.44321300 1.24630800

H 2.95246400 -0.20740900 -0.10505000

H 1.21052400 -0.89033400 -1.71981900

H 1.37589100 -2.14590000 -0.49754100

H -0.26594500 1.15697600 1.43790900

H -0.45920600 2.42494700 0.22078300

H 1.56919200 1.64611900 -0.97266600

F -2.26416200 0.51740900 -0.15672600

H -0.80823800 0.73329800 -1.54110300

H -1.01745600 -1.64561100 -0.98993200

F -0.65649900 -1.41986200 0.98148400

G^0 = -434.403537 hartrees

Diequatorial *trans*-1,2-difluorocyclohexane (DMSO – SMD)

0 1

C 0.60496600 1.46983800 0.10719200

C 1.87307600 0.70591800 -0.29710300

C 1.87307600 -0.70591800 0.29710300

C 0.60496600 -1.46983800 -0.10719200

C -0.63867800 -0.69529300 0.29438100

C -0.63867900 0.69529300 -0.29438100

H 2.75270800 -1.26235200 -0.03644000

H 1.92917400 0.64225000 -1.38985100

H 2.75270800 1.26235200 0.03643900

H 0.58899100 1.61296300 1.19311700

H 0.57321900 2.45699000 -0.36008500

H 0.58899200 -1.61296300 -1.19311700

H 0.57321900 -2.45699000 0.36008500

H 1.92917400 -0.64225000 1.38985100

F -1.79221400 1.39187600 0.17835100

F -1.79221400 -1.39187600 -0.17835100

H -0.75034700 -0.64213900 1.37998600

H -0.75034700 0.64213900 -1.37998600

G^0 = -434.404982 hartrees

Diaxial *cis*-1,3-difluorocyclohexane (DMSO – SMD)

0 1

C -1.26282400 1.11545500 0.19632100

C 0.00000100 1.57725400 -0.53801200

C 1.26282500 1.11545400 0.19632100

C 1.27958500 -0.38473000 0.42649300

C 0.00000000 -0.91885500 1.04954800

C -1.27958500 -0.38472900 0.42649300

H 2.16668300 1.40466000 -0.34656100

H 0.00000100 1.18221700 -1.55841800

H 0.00000100 2.66693100 -0.62277300

H -1.31374900 1.59187400 1.18235900

H -2.16668200 1.40466100 -0.34656100

H 2.14286000 -0.68220800 1.02349500

H 1.31375000 1.59187300 1.18235900

H -0.00000100 -0.61355800 2.10158900

H -2.14286100 -0.68220600 1.02349500

F -1.47537100 -1.03176200 -0.84543700

F 1.47537000 -1.03176300 -0.84543700

H -0.00000100 -2.01160700 1.03589200

G^0 = -434.406060 hartrees

***Trans*-1,3-difluorocyclohexane (DMSO – SMD)**

0 1

C -1.47988200 0.76764600 0.57673100

C -0.55850700 1.62330200 -0.29953300

C 0.91841400 1.27936200 -0.05469500

C 1.14487500 -0.19830300 -0.30023300

C 0.26621900 -1.07697900 0.57336700

C -1.20477000 -0.71747000 0.42746000

H 1.56616200 1.86732400 -0.71006400

H -0.80051300 1.46473400 -1.35557500

H -0.72925700 2.68237100 -0.09260800

H -1.32604700 1.01710400 1.63267300

H -2.53173800 0.96196500 0.35081100

H 1.18661800 1.50909400 0.98278400

H 0.54126600 -0.92177900 1.62197800

H -1.81395500 -1.30688400 1.11327700

F -1.62878700 -1.12321600 -0.88918700

H 0.41660100 -2.13312200 0.33766500

H 1.01876600 -0.44080500 -1.35730600

F 2.51256600 -0.51737900 -0.00661600

G^0 = -434.407055 hartrees

Diequatorial *cis*-1,3-difluorocyclohexane (DMSO – SMD)

0 1

C 1.26961000 1.06685700 -0.23477100

C 0.00000000 1.82256500 0.18521100

C -1.26961000 1.06685700 -0.23477100

C -1.24244700 -0.33748000 0.33428000

C 0.00000000 -1.10980500 -0.08255000

C 1.24244700 -0.33748000 0.33428000

H -2.16260600 1.59107400 0.11473000

H 0.00000000 1.96286400 1.27194500

H 0.00000000 2.81860600 -0.26230800

H 1.31920300 1.00190700 -1.32715800

H 2.16260600 1.59107400 0.11473000

H -1.31920300 1.00190700 -1.32715900

H 0.00000000 -1.22161100 -1.17164300

H 0.00000000 -2.10223400 0.37410700

H -1.34345200 -0.31839400 1.42211900

F -2.38770700 -1.05755000 -0.13564200

F 2.38770700 -1.05754900 -0.13564200

H 1.34345200 -0.31839500 1.42211900

G^0 = -434.406722 hartrees

Diaxial *trans*-1,4-difluorocyclohexane (DMSO – SMD)

0 1

C 0.52727300 -1.26650200 0.55687300

C -0.52727300 -1.26650200 -0.55687400

C -1.36314100 -0.00000200 -0.56459900

C -0.52727600 1.26650100 -0.55687700

C 0.52727300 1.26650200 0.55687500

C 1.36313900 -0.00000200 0.56459800

H -2.07198800 -0.00000500 -1.39375500

H -0.04120600 -1.33710800 -1.53517100

H -1.18167700 -2.13684100 -0.46410200

H 0.04120800 -1.33711200 1.53517000

H 1.18168000 -2.13683900 0.46409700

H 0.04120200 1.33711500 1.53516900

H 1.18168100 2.13683700 0.46409100

H -0.04120400 1.33711200 -1.53517000

H -1.18168300 2.13683700 -0.46409400

F -2.18458600 0.00000100 0.62317800

F 2.18459000 0.00000200 -0.62317500

H 2.07198100 -0.00000300 1.39375800

G^0 = -434.40799 hartrees

***Cis*-1,4-difluorocyclohexane (DMSO – SMD)**

0 1

C -0.58384800 1.25945200 0.08980700

C 0.79999800 1.26698200 -0.57724400

C 1.58631000 0.00000000 -0.29267400

C 0.79999800 -1.26698200 -0.57724400

C -0.58384700 -1.25945200 0.08980700

C -1.33694800 0.00000000 -0.29058700

H 2.53783300 0.00000000 -0.82604500

H 0.69055100 1.34037700 -1.66481300

H 1.37524300 2.13791600 -0.25451000

H -0.47604000 1.28855400 1.17859300

H -1.15054800 2.14276400 -0.21437600

H -0.47603900 -1.28855400 1.17859300

H -1.15054700 -2.14276400 -0.21437500

H 0.69055100 -1.34037600 -1.66481400

F -2.60152700 0.00000000 0.38924700

H -1.58553300 0.00000000 -1.35533800

H 1.37524300 -2.13791600 -0.25451200

F 1.94367200 0.00000000 1.10413200

G^0 = -434.408066 hartrees

Diequatorial *cis*-1,4-difluorocyclohexane (DMSO – SMD)

0 1

C -0.74703400 1.26179100 0.17982500

C 0.74703500 1.26179000 -0.17982900

C 1.40854300 0.00000100 0.34366500

C 0.74703700 -1.26179200 -0.17983000

C -0.74703700 -1.26179200 0.17982600

C -1.40854500 0.00000100 -0.34366600

H 0.87000400 1.29552800 -1.26779600

H 1.23402000 2.14440500 0.24087100

H -0.87000400 1.29553200 1.26779200

H -1.23401800 2.14440500 -0.24087700

H -0.87000200 -1.29553200 1.26779400

H -1.23401800 -2.14440400 -0.24088600

H 0.87000300 -1.29553000 -1.26779800

H 1.23401800 -2.14440500 0.24088000

H -1.43726200 -0.00000200 -1.43627200

H 1.43725400 -0.00000200 1.43627200

F 2.78139400 0.00000100 -0.06971200

F -2.78139300 0.00000100 0.06972000

G^0 = -434.407573 hartrees

Dimer diaxial trans-1,2-difluorocyclohexane

0 1 0 1 0 1
C(Fragment=1) -2.09948900 -0.74796600 1.15158900
C(Fragment=1) -2.78890300 0.56970100 0.85074700
C(Fragment=1) -2.46380200 1.11712900 -0.53944200
C(Fragment=1) -2.61955500 0.08298800 -1.63936600
C(Fragment=1) -1.88060900 -1.22281400 -1.32023200
C(Fragment=1) -2.31188700 -1.78316900 0.04006100
H(Fragment=1) -3.06581200 2.00690100 -0.73519200
H(Fragment=1) -2.55950100 1.32546400 1.60556100
H(Fragment=1) -1.02969900 -0.54808400 1.26096700
H(Fragment=1) -2.46008300 -1.11845800 2.11501700
H(Fragment=1) -3.69213700 -0.11219900 -1.74741100
H(Fragment=1) -2.27559300 0.51849500 -2.58165100
H(Fragment=1) -2.07271000 -1.95291100 -2.11129100
H(Fragment=1) -0.80184200 -1.04363500 -1.30810000
H(Fragment=1) -3.36853700 -2.06904900 0.00368000
H(Fragment=1) -1.74161300 -2.68545400 0.27315100
C(Fragment=2) 2.61891500 -0.08685300 1.63924400
C(Fragment=2) 1.88133400 1.22045400 1.32311700
C(Fragment=2) 2.31375600 1.78380300 -0.03556600
C(Fragment=2) 2.10040700 0.75164000 -1.14973800
C(Fragment=2) 2.78844700 -0.56747800 -0.85211100
C(Fragment=2) 2.46279800 -1.11812800 0.53667300
H(Fragment=2) 1.74465500 2.68737300 -0.26656200
H(Fragment=2) 0.80238600 1.04231800 1.31005400
H(Fragment=2) 2.07375600 1.94833800 2.11613300
H(Fragment=2) 3.69161600 0.10713100 1.74828000
H(Fragment=2) 2.27415000 -0.52444300 2.58027100
H(Fragment=2) 1.03037800 0.55336100 -1.25966000
H(Fragment=2) 2.46152600 1.12416000 -2.11218400
H(Fragment=2) 2.55835700 -1.32113900 -1.60881100
H(Fragment=2) 3.06436900 -2.00865800 0.73032200
H(Fragment=2) 3.37073300 2.06832300 0.00190900
F(Fragment=2) 4.19226600 -0.38619500 -0.87968000
F(Fragment=2) 1.11326400 -1.55295000 0.48642200
F(Fragment=1) -1.11443400 1.55269500 -0.49050300
F(Fragment=1) -4.19252600 0.38715300 0.87883500

H^0 = -868.707573 hartrees

Dimer diaxial cis-1,3-difluorocyclohexane

0 1 0 1 0 1
C(Fragment=1) -2.20414100 -0.00057900 1.74394000
C(Fragment=1) -1.72157500 -1.26330100 1.02205500
C(Fragment=1) -2.15482500 -1.28368100 -0.43661400
C(Fragment=1) -1.81128800 -0.00070900 -1.18270800
C(Fragment=1) -2.14872000 1.28368100 -0.43624400
C(Fragment=1) -1.71736200 1.26103100 1.02293700
H(Fragment=1) -1.71339300 -2.13876300 -0.95477300
H(Fragment=1) -2.09723300 -2.16502900 1.51311200
H(Fragment=1) -3.29656900 0.00111500 1.78149300
H(Fragment=1) -1.84803500 -0.00148000 2.77829800
H(Fragment=1) -0.73160500 -0.00392700 -1.35811800
H(Fragment=1) -2.30609700 0.00077300 -2.15696900
H(Fragment=1) -1.70246000 2.13671500 -0.95360400
H(Fragment=1) -0.62345900 1.31077000 1.03995000
H(Fragment=1) -2.09121800 2.16358400 1.51387300
C(Fragment=2) 2.92237600 -1.26623400 0.89100000
C(Fragment=2) 2.37817100 -0.00423300 1.56857200
C(Fragment=2) 2.92449100 1.25991300 0.89667500
C(Fragment=2) 2.63402000 1.28295200 -0.59683000
C(Fragment=2) 3.04818200 0.00172200 -1.31189000
C(Fragment=2) 2.63180500 -1.28201000 -0.60257100
H(Fragment=2) 2.50532000 2.16091500 1.35125800
H(Fragment=2) 1.28708500 -0.00321300 1.51377800
H(Fragment=2) 2.64139900 -0.00682500 2.62914300
H(Fragment=2) 4.01033800 -1.32083200 1.02093300
H(Fragment=2) 2.50172900 -2.16859200 1.34151900
H(Fragment=2) 3.10975200 2.14470500 -1.07183400
H(Fragment=2) 2.65536500 0.00435100 -2.33119500
H(Fragment=2) 3.10598700 -2.14241400 -1.08154800
H(Fragment=2) 4.01253400 1.31209900 1.02692800
F(Fragment=2) 1.23873500 -1.47371200 -0.78311400
F(Fragment=1) -3.55583700 -1.49432100 -0.48185700
H(Fragment=2) 4.14167900 0.00088500 -1.38822300
F(Fragment=2) 1.24131800 1.47790400 -0.77657100
H(Fragment=1) -0.62774800 -1.31579200 1.03740500
F(Fragment=1) -3.54868100 1.50120000 -0.48305500

H^0 = -868.709179 hartrees

Dimer diaxial trans-1,4-difluorocyclohexane

0 1 0 1 0 1
C(Fragment=1) -2.55036800 -1.01533300 1.26679500
C(Fragment=1) -2.11588900 -1.73415600 -0.00015200
C(Fragment=1) -2.55003600 -1.01507800 -1.26705800
C(Fragment=1) -2.14092900 0.46349100 -1.26490600
C(Fragment=1) -2.59123000 1.17618200 0.00011700
C(Fragment=1) -2.14114400 0.46320200 1.26504500
H(Fragment=1) -2.13243000 -1.52918600 -2.13650400
H(Fragment=1) -2.45987200 -2.77112600 -0.00032100
H(Fragment=1) -3.63975000 -1.09666700 1.33558400
H(Fragment=1) -2.13304700 -1.52965100 2.13625200
H(Fragment=1) -1.05400300 0.55907800 -1.32158300
H(Fragment=1) -2.55868200 0.97341500 -2.13644500
H(Fragment=1) -2.25712900 2.21521900 0.00026500
H(Fragment=1) -1.05421100 0.55867000 1.32182400
H(Fragment=1) -2.55891000 0.97298900 2.13665700
C(Fragment=2) 2.55019500 1.01525200 -1.26688200
C(Fragment=2) 2.14110700 -0.46332300 -1.26496100
C(Fragment=2) 2.59134000 -1.17616400 -0.00000400
C(Fragment=2) 2.14103500 -0.46337900 1.26496300
C(Fragment=2) 2.55015000 1.01519800 1.26695800
C(Fragment=2) 2.11580700 1.73414400 0.00003700
H(Fragment=2) 2.25731400 -2.21522600 -0.00003200
H(Fragment=2) 1.05418100 -0.55888700 -1.32167100
H(Fragment=2) 2.55887900 -0.97314100 -2.13655000
H(Fragment=2) 3.63956200 1.09668900 -1.33580900
H(Fragment=2) 2.13273200 1.52945100 -2.13634300
H(Fragment=2) 2.55873800 -0.97324100 2.13656000
H(Fragment=2) 2.13265700 1.52937300 2.13641500
H(Fragment=2) 2.45966100 2.77115600 0.00006400
F(Fragment=2) 0.68868500 1.81562800 0.00002700
H(Fragment=2) 3.63951700 1.09659700 1.33592300
F(Fragment=1) -4.01585700 1.22602100 -0.00001400
F(Fragment=2) 4.01595500 -1.22589300 0.00002800
H(Fragment=2) 1.05410200 -0.55893700 1.32159500
H(Fragment=1) -3.63939400 -1.09648400 -1.33618900
F(Fragment=1) -0.68879900 -1.81578900 0.00002400

H^0 = -868.718046 hartrees
